# Evaluation of a Novel Mucosal Administered Subunit Vaccine on Colostrum IgA and Serum IgG in Sows and Control of Enterotoxigenic *Escherichia coli* in Neonatal and Weanling Piglets: Proof of Concept

**DOI:** 10.3389/fvets.2021.640228

**Published:** 2021-02-11

**Authors:** Maria Fernanda Jabif, Emanuel Gumina, Jeffrey W. Hall, Xochitl Hernandez-Velasco, Sherry Layton

**Affiliations:** ^1^Vetanco S.A., Buenos Aires, Argentina; ^2^Vetanco USA, Inc., Saint Paul, MN, United States; ^3^Departamento de Medicina y Zootecnia de Aves, Facultad de Medicina Veterinaria y Zootecnia, Universidad Nacional Autonoma de Mexico, Ciudad de Mexico, Mexico

**Keywords:** colibacillosis, IgA, mucosal vaccine, post-weaning diarrhea, subunit vaccine

## Abstract

The purpose of the present study was to evaluate the ability of a novel experimental subunit vaccine (ESV), induce colostrum IgA and serum IgG in sows, and to control enterotoxigenic *Escherichia coli* (ETEC) disease in neonatal and weanling piglets. The vaccine was tested in three experiments. Experiment 1 consisted of two independent trials. In each trial, 20 pregnant sows/groups were vaccinated intramuscularly (IM) with a commercial *E. coli* vaccine or intranasally with ESV at weeks 11 and 13 of pregnancy. Blood and serum samples were obtained within 12 h post-partum. In Experiment 1, intranasal vaccination with ESV significantly increased the sample-to-positive (S/P) ratio of secretory IgA in the colostrum of sows (*P* < 0.01, trial 1; *P* < 0.05, trial 2) compared to the IM vaccine. In Experiment 2, twenty-five 3-day old piglets were randomly allocated into two groups, control (*n* = 13) or ESV (*n* = 12) and were oral gavaged with the respective treatments on days 3 and 14 of life. On days 17–19, all piglets were challenged using a mixed ETEC culture via oral gavage. Within 72 h, all control group animals developed disease consistent with colibacillosis. Conversely, the ESV treated group remained disease free over the 7-day observation period and had significant increases in body weight gain compared to the control group piglets. In Experiment 3, thirty 28-day old piglets were randomly allocated, control (*n* = 15) or ESV (*n* = 15), and on days 33 and 43 of life, piglets were either given by oral gavage 2.0 mL saline (control group) or 2.0 mL ESV. At days 46 and 47 of life, all pigs were challenged with a mixed culture of ETEC and observed for clinical signs of disease. Results of Experiment 3 were similar to those observed in Experiment 2. This study indicates the ESV can induce better levels of colostrum secretory IgA in pregnant sows than IM vaccination, which may be protective to neonatal piglets. Further, the vaccine can protect piglets as early as 3 days of age from an ETEC infection. Importantly, the data suggest a single vaccine could be used across the farrowing, suckling, and weaning program to protect against pathogenic *E. coli*.

## Introduction

The development of vaccines for effective control of foodborne pathogens and infection represents a significant development in reducing public health risk (1). Advancements in biotechnology have increased innovative potential and allow new technologies to be used as a promising control strategy for alternatives to antibiotics (2), and vaccines are highly regarded for their perceived feasibility and effectiveness (3).

Pathogenic *E. coli* infections or colibacillosis is one of the most prevalent diseases affecting the global swine industry (4). Enterotoxigenic *Escherichia coli* (ETEC) is a significant cause of illness and death in neonatal and recently weaned pigs; in some cases, young pigs can lose up to 40% of their body weight, and in severe cases, mortality can reach 100% (5–8). Colibacillosis has a direct economic impact on producers and represents a potential human transmission route of foodborne illness. Standard treatment options often include incorporating antibiotics to control and limit the spread of the disease; however, the condition is becoming increasingly challenging to treat due to acquired antibiotic resistance (9). Moreover, consumer pressure and changing government regulations may limit or omit antibiotics necessitating the need for alternative intervention strategies (10).

Enterotoxigenic *E. coli* produce two main pathogenic determinants; fimbria adhesins and toxins. ETEC fimbriae promote initial bacterial adherence to epithelial cell receptors and ultimately allow the ETEC to colonize the small intestine. ETEC fimbriae most commonly associated with neonatal diarrhea are F4, F5, F6, and F41, while F4 and F18 are most common among ETEC-induced post-weaning cases diarrhea (11). Once close to the intestinal epithelium, ETEC produces one or more toxins, such as heat stable toxin a (STa), heat stable toxin b (STb), and/or heat labile toxin (LT), which enter host cells and disrupt fluid homeostasis. The result is electrolyte-rich fluid secretion and diarrhea, leading to weight loss, slow growth, and possibly death (4, 11, 12).

For neonatal piglets, protection from ETEC infection primarily relies on sow vaccination and passive colostrum antibody immunity. Here, sows are commonly vaccinated against one or more types of ETEC fimbriae. Due to various issues, including host genetics and the heterogenic distribution and antigenicity of fimbriae, protection is often incomplete and veins as the piglets age and ceases upon weaning (12). Unfortunately, there are no vaccines currently available that effectively protect against post-weaning colibacillosis (12). A significant amount of research has developed effective pathogenic *E. coli* vaccines, but much of this research has targeted the diverse array of fimbriae and toxins produced by these strains. These vaccines often lack cross-protection between strains or require conjugation to other potent immune system stimulating molecules to induce adequate protection (4, 13–16). This leaves the swine population at risk of infection from strains not included in the vaccine and may require complex or economically unfeasible vaccine production processes.

We have been working to create a novel vaccine platform that incorporates a subunit/epitope sequence, common to all ETEC strains (broad-spectrum), into an inactivated orally administered vaccine platform that protects against infection and disease by inducing mucosal immunity.

The mucous membranes constitute the primary portal of entry for infectious agents. They include membranes of the nasal, respiratory, gastrointestinal, and genitourinary tract and the ocular conjunctiva, the inner ear, and the ducts of all exocrine glands. Collectively they cover more than 400 m^2^ in humans, compared to only 2 m^2^ of skin, and serve as the first line of defense against infection at the entry points for a variety of pathogens, including pathogenic *E. coli*. (2, 17–20). The gastrointestinal system is the largest lymphoid organ in the body containing an estimated 70 to 80% of the body's immunoglobulin–producing cells (21). Over 80% of all the activated B cells in the body are located at the mucosal tissues (22).

Despite its essential role, currently, only a handful of vaccines specifically target this area of the immune system despite strong evidence that a robust mucosal response can effectively prevent systemic infections (17). Increasing evidence has indicated that mucosal vaccination can induce systemic and local mucosal immunity, while systemic immunization generally fails to elicit strong mucosal immunity (23). Also, the concept of a standard mucosal immune system predicts that induction of immunity at one mucosal surface, such as the gut, can provide immunity at another mucosal surface, such as the lung (24), providing a vital link for immunity transfer throughout mucosal surfaces. Mucosal immunity may prove to be the link in fighting a complicated infection in which systemic and local immunity is necessary for preventing the spread and transmission of infectious disease and foodborne pathogens (25).

Our vaccine development, in which a single vaccine, can simultaneously and effectively control all or most serotypes/strains that make up the 150–200 serotypes that represent the *E. coli* family of pathogens (broad spectrum) and provide protection in multiple species (swine, poultry, bovine, fish, humans) potentially represent practical biotechnological progress as an alternative intervention strategy in controlling diseases and foodborne pathogens. Hence, the purpose of the present study was to evaluate this novel mucosal administered subunit vaccine on the induction of colostrum IgA and serum IgG in sows and to prevention of colibacillosis in neonatal and weanling piglets.

## Materials and Methods

### Development of the Experimental Subunit Vaccine

Briefly, vaccine construction was as follows. A synthetic DNA sequence, coding for an antigenic epitope common to a broad-spectrum of *E. coli* serotypes was inserted into the multiple cloning sites of the plasmid by direct ligation into a *E. coli*-*Bacillus subtilis* shuttle expression plasmid. The genetic sequence was put under the control of an IPTG inducible promoter present on the expression plasmid. The modified expression plasmid was then transfected into *E. coli* TOPO 1 cells for confirmation of gene insert and multiplication of the plasmid. Once plasmid insertion was confirmed by colony PCR, DNA sequencing was performed to verify the insert sequence was correct. The multiplied confirmed plasmid was then isolated, concentrated, and transfected into *Bacillus subtilis* VBTSLL11™, a proprietary *Bacillus* strain explicitly selected for use in the Biotech Vac platform. Expression of the epitope is under the control of an IPTG inducible promoter present on the expression plasmid. Production of the epitope was confirmed using SDS-PAGE and western blotting. This newly constructed and verified *Bacillus* strain was used to manufacture the antigenic *E. coli* subunit. The bacteria were cultured in Tryptic Soy Broth (TSB) at 37°C with slight agitation. After 4 h of growth, the culture was induced with 1 mM of Isopropyl β-D-1-thiogalactopyranoside (IPTG) followed by an additional 5 h of incubation. Once fermentation was complete, the culture was inactivated with formaldehyde (final conc. 0.1% v/v) and added to a proprietary encapsulation media to incorporate the epitopes into micro-particles for delivery. The encapsulation media is comprised of monomeric and polymeric carbohydrates at specific ratios and concentrations, which form the micro-particles during formulation. This matrix protects the subunit as it transits the stomach and dissolves as it moves through the small intestine, eventually delivery the antigenic subunit to antigen presenting cells in the Peyer's patches of the small intestine. The concentration of the antigenic subunit is calculated using a standard direct ELISA assay with a standard curve created using synthetic subunit. Subunit concentration for each 2.0 mL dose of vaccine is ~500 ng.

### Animal Use Protocol and Diets

In the present study, all experiments were conducted in an integrated commercial farm in Cordoba, Argentina. In each experiment, all animal handling procedures followed the Institutional Committee's guidelines of use and care of experimental animals of the National Institute of Agronomic Technologies (INTA). All diets were formulated to meet or exceed the National Research Council (26) nutrient requirements, respectively.

### Experiment 1. Evaluation of the Induction of Secretory IgA in Colostrum and Serum IgG in Sows

#### Animal Source and Housing Conditions

This study was conducted to evaluate the induction of secretory IgA and serum IgG in sows vaccinated with either a commercial intramuscular *E. coli* vaccine or intranasal administration of ESV in two independent trials.

The farm has 1,000 sows in an integrated system using Pig Improvement Company (PIC®) genetics (Landrace × Yorkshire) for the complete cycle. Weaned piglets are moved to other units. Artificial insemination is performed in its entirety, with liquid genetics purchased at PIC® from boars line 337. The farm provides 2.5 m^2^/sow in its gestation facilities. The general management of the females is to keep them individually until 40 days of gestation, where the pregnancy is confirmed by ultrasound. They go to group gestation until they enter the maternity room. Sows are admitted to farrowing at 110 days gestation and are housed in the same room. Deliveries are not hormonally scheduled; they are natural since there are workers on the night shift to attend to them. Lactation period lasts 24 days on average, after which the animals are weaned. Females have a microchip in the ear to access their physiological information (Age, number of deliveries, days of gestation, vaccines received, previous treatments or pathologies, reproductive data). Feeding is with the sorter automatic feeding system. The female enters a crate of her own free will that reads her chip and administers the stipulated amount of food according to nutritional requirements.

#### Experimental Design

In each trial, 40 pregnant sows were randomly divided into two experimental groups (*n* = 20 sows). Table 1 shows the number of deliveries and age of the pregnant sows selected for the evaluation of the induction of secretory IgA in colostrum and serum IgG in Experiment 1. The Control group from each trial was vaccinated intramuscularly with a commercial *E. coli* vaccine according to the label. The other group from each trial was intranasally vaccinated with 2 mL of Biotech Vac *E. coli*. Each sow was vaccinated with the respective treatment vaccine at weeks 11 and 13 of pregnancy. Blood (collected in serum separator tubes) and colostrum samples were obtained within the first 12 h postpartum and analyzed by ELISA to quantify secretory IgA in colostrum and IgG in the serum as described below.

**Table 1:** Number of deliveries and age of the pregnant sows selected for the evaluation of the induction of secretory IgA in colostrum and serum IgG in Experiment 1.

	**Trial 1**		**Trial 2**	
	**Vaccinated intramuscularly with a commercial *E. coli* vaccine**	**Intranasally vaccinated with ESV**	**Vaccinated intramuscularly with a commercial *E. coli* vaccine**	**Intranasally vaccinated with ESV**
Number of deliveries	3.09 ± 0.19	3.10 ± 0.20	3.06 ± 0.12	3.05 ± 0.18
Age in days	641.11 ± 28.44	665.21 ± 28.90	662.30 ± 18.84	649.80 ± 27.85

*Data expressed as mean ± SE. N = 20; P > 0.05*.

#### Antigen-Specific ELISA

For the quantification of antigen-specific IgG and secretory IgA, serum was separated from the other blood components by centrifugation after letting the blood clot at room temperature overnight. Colostrum was separated from the milk's fat portion by centrifugation for 60 min at 149,000 × g at 4°C. The colostrum separates into three distinct fractions, fat (top), casein (pellet), and whey (middle clear liquid) fractions, respectively. Greater than 90% of the immunoglobulins are in whey, this was recovered by piercing the fat layer with a Pasteur pipette and drawing up the whey (middle) layer Synthetic antigen homologous to the ESV subunit (50 ng/mL) was absorbed in wells of an immunosorp 96-well plate (Nunc) overnight at 4°C. The following morning, the antigen solution was removed, and wells were blocked with 300 μl of Superblock BSA (Bio = Rad Laboratories) for 1 h at room temperature. Serum (1:400) and colostrum whey (1:50) samples were added to the plate in triplicate and incubated for 1 h at room temperature; the plate was then washed 3X with Washing Buffer (Bethyl Laboratories). The secondary antibody was next added to each sample and control well: serum sample wells were incubated with goat anti-pig IgG-HRP (Bethyl Laboratories, 1 mg/mL, 1:50,000 dil.) or goat anti-pig IgA-HRP (Bethyl Laboratories, 1 mg/ml, 1:40,000 dil.), respectively for 1 h at room temperature. The plate was washed 3 times with Washing Buffer to develop the plate, 100 μl of 1-C TMB Substrate (Bethyl Laboratories) was used according to manufacturer's instructions and allowed to develop for 3 min. The HRP reaction was stopped with 100 μl of diluted 0.16 M sulfuric acid. The absorbance at 450 nm of each well was read using a spectrophotometer (Epoch). The average absorbance obtained from the positive, negative, and sample controls was used to calculate the sample-to-positive (S/P) ratios.

### Experiment 2. Evaluation of ESV to Control Enterotoxigenic *Escherichia coli* in Neonatal Piglets

#### Animal Source and Husbandry

The farrowing house has weekly rooms that are filled with sows with farrow dates in the same week with a capacity of 12 farrowing pens per room. Each farrowing pen is separated by plastic walls ~50 cm high in its entirety, preventing contact between piglets from different farrowing pens. Parturitions are scheduled at day 113. Newborn piglets are dried with paper; their umbilical cord is tied, and it is disinfected with iodine. There is no fang, or tail, cut. The farrowing piglets have a thermal blanket to maintain their body temperature. This blanket will be adjusted as the piglets are older (week of birth: 28°C; week 2: 26°C; week 3: 24°C). Weaning is carried out with an average day of 21 and 6 kg of weight. The piglet sanitary scheme consists of coccidiostat, 1 mL (Diclazuril) on day 1. On day 3, Iron dextran two mL (IM) and *Mycoplasma* vaccine at 70 and 90 days of life. For the trial, piglets that did not manifest unknown digestive, respiratory disease, or locomotion problems, were randomly selected. As the *E. coli* challenge was carried out in the same room where there were other piglets, the measures taken to avoid spreading *E. coli* were: On the day of inoculation, the rest of the piglets that did not participate in the trial were given a dose of free acid Ceftiofur 0.2 ml / piglet, a broad spectrum antibiotic with a depot action of 7 days. Specially colored footwear, gloves, and clothing were utilized when working in the farrowing pens. All piglets were identified by ear tag number.

#### Experimental Design

In this experiment, twenty-five 3-day old piglets were randomly allocated in one of two experimental groups. A non-treated control group (*n* = 13) that received 2.0 mL of saline by oral gavage on days 3 and 14 of life or a treated group (*n* = 12) that received 2.0 mL of ESV vaccine by oral gavage on days 3 and 14 of life. All piglets in both groups were challenged with 2.0 mL of ETEC *E. coli* by oral gavage (challenge preparation described below) on days 17, 18, and 19 of life. The clinical response of each piglet was monitored throughout the experiment in terms of occurrence of diarrhea, fecal consistency score, and morbidity for 7 days following *E. coli* challenge. The fecal score is described below. Additionally, individual weight gain was determined. During the course of the experiment, neither the piglets nor the lactating sows received antibiotic treatment, and antibiotics were not present in the commercial feed.

#### Challenge With Enterotoxigenic Escherichia Coli

Two wild-type Enterotoxigenic *E. coli* (ETEC) field isolates, VBTEColi-1 and VBTEColi-2, originally isolated from swine farms in Argentina were grown individually to log phase, combined, serially diluted, and enumerated by spectrophotometric density and comparison to a previously generated standard curve. Combined, these strains produce LT and STb toxins and the K88 and F18 fimbriae.

These strains were diluted to ~10^8^ cfu/mL for the challenge by oral gavage at a dose of 2.0 mL/pig for the number of consecutive days as described above and below.

### Experiment 3. Evaluation of ESV to Control Enterotoxigenic *Escherichia coli* in Weanling Piglets

#### Animal Source and Husbandry

The experimentation unit was 80 meters away from the farm. It was a mobile unit with a capacity for 100 pigs up to 25 kg. Sidewalls of double curtain with UV protection plus a stainless steel mesh, the ceiling was made of plastic sheet with insulating material inside, the floor was plastic slats with collection of fecal matter in trays, with waste form the experimental unit collected by a pathogenic waste company and disposed of off-site. The unit had a 400-liter tank for drinking water and two feeders (One in each pen). The piglets were weaned at an average of 21 days of life and taken to this unit, conditioned at 28°C, and commercial pre-starter feed in floor presentation was available.

#### Experimental Design

In this experiment, thirty 28-day-old piglets were randomly assigned to either a non-treated control group or a ESV vaccine treated group (*n* = 15/group), transferred to adjacent weaning boxes isolated from the rest of the commercial farm, and allowed to acclimate for 5 days. Following the acclimation period (day 33 of life), pigs were either given by oral gavage 2.0 mL saline (control group) or 2.0 mL Biotech Vac *E. coli*. Subsequently, the same treatment was administered 10 days (day 43 of life) following the first administration. Three and four days following the second treatment administration (day 46 and 47 of life), all pigs in both groups were challenged with 2.0 mL of ETEC by oral gavage (challenge preparation described above). Pigs in both groups were observed for 10 days following the challenge. The clinical response of each piglet was monitored throughout the experiment in terms of occurrence of diarrhea, fecal consistency score, and morbidity. Also, individual weight gain was measured. Pigs were fed standard commercial diets containing no antibiotics.

### Fecal Scoring

In each experiment, subjective diarrhea scores were recorded daily by the same person and were based on the following: 1, well-formed feces; 2, mild diarrhea; 3, severe diarrhea (27). Scores were recorded on a pen basis following observations of individual pigs and signs of stool consistency. The score is reported as the number of piglets with diarrhea score/number of piglets in the group (%).

### Statistical Analysis

Body weights and S/P ratio were subjected to one-way ANOVA as a completely randomized design using the GLM procedure of SAS (28). Treatment means were partitioned using Duncan's multiple range test at α <0.05 and α <0.01 indicating statistical significance. Fecal scoring was compared by a chi-square test of independence (29) to determine the significance at α <0.001.

## Results

**Figure 1:**
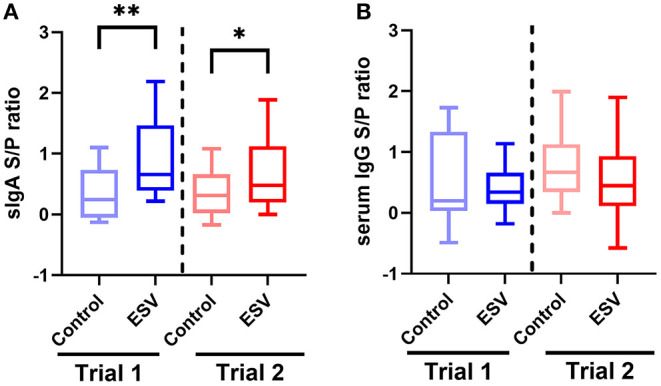
ESV antigen-specific ELISA S/P ratios from vaccinated pregnant sows in Experiment 1. **(A)** Secretory IgA S/P ratio in colostrum. **(B)** IgG S/P ratio in serum. Control—commercial IM *E. coli* vaccination. Experimental subunit vaccine (ESV)—intranasal vaccination. *P < 0.05 and **P < 0.01 (*n* = 20 sows/group/trial).

Figure 1 shows the evaluation of secretory IgA induction in colostrum and serum IgG in sows vaccinated with either a control commercial intramuscular *E. coli* vaccine or intranasal administration of ESV in two independent trials of Experiment 1. In both trials, intranasal vaccination with ESV significantly elevated the S/P ratio of antigen-specific secretory IgA in the colostrum of sows (*P* < 0.01 trial 1; *P* < 0.05 trial 2) sampled within 12 h postpartum as compared to the control group (Figure 1A). Interestingly, the antigen-specific serum IgG S/P ratios were similar (*P* > 0.05) between the treatment and trial groups (Figure 1B).

**Figure 2:**
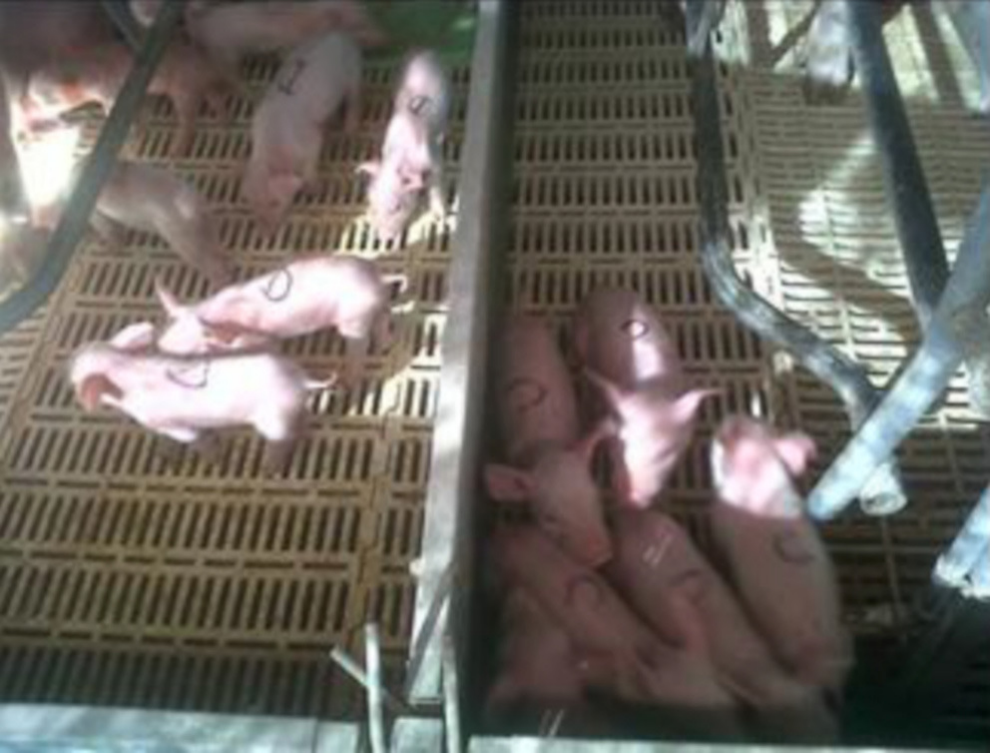
Visual representation of colibacillosis clinical signs and associated diarrhea in Experiment 2. Piglets in the ESV vaccinated group displayed normal behavior and were absent of clinical signs **(Left)**, while piglets in the Control group huddled together and were lethargic **(Right)**.

The evaluation of ESV against ETEC on diarrhea scores of neonatal piglets displaying colibacillosis in Experiment 2 are shown in Figure 2 and Table 2. Forty-eight hours after challenge, 100% (13/13) of the piglets in the saline control group had developed clinical signs, including lethargy, hollow flanks, anorexia, and diarrhea consistent with colibacillosis, with diarrhea continuing for 72 h. In contrast, the piglets that were treated with ESV exhibited no clinical signs and did not develop diarrhea (*P* < 0.001) throughout the 7-day observation period (Figure 2 and Table 3). In this experiment, piglets in both groups started with the same average body weight. However, 7 days post *E. coli* challenge, piglets in the ESV treated group had a significant (*P* < 0.05) increase in final body weight and body weight gain when compared to the saline control piglets (Table 4). No mortality was observed in either treatment group.

**Table 2:** Evaluation of an experimental subunit vaccine (ESV) orally administered (Biotech Vac *E. coli*) against enterotoxigenic *E. coli* (ETEC) on diarrhea scores of neonatal piglets displaying colibacillosis.

**Days post-challenge**	**Score**	**Control**	**ESV**
1	1	13/13 (100%)	12/12 (100%)
2	1	13/13 (100%)	12/12 (100%)
3	3	13/13 (100%)	0/12 (0%)^*^
4	2	13/13 (100%)	0/12 (0%)^*^
5	2	13/13 (100%)	0/12 (0%)^*^
6	1	13/13 (100%)	12/12 (100%)
7	1	13/13 (100%)	12/12 (100%)

*Experiment 2. Data are expressed as the number of piglets with diarrhea score/number of piglets in the group (%)*.

*Diarrhea scores: 1, well-formed feces; 2, mild diarrhea; 3, severe diarrhea*.

* *Asterisk within rows indicates a significant difference at P < 0.001*.

**Table 3:** Evaluation of an experimental subunit vaccine (ESV) against enterotoxigenic *E. coli* (ETEC) on initial body weight, final body weight (day 26), and body weight gain of neonatal piglets.

	**Control**	**ESV**
Initial body weight	1.64 ± 0.04^a^	1.62 ± 0.04^a^
Final body weight	5.45 ± 0.08^b^	6.22 ± 0.07^a^
Body weight gain	3.81 ± 0.09^b^	4.60 ± 0.07^a^

*Experiment 2. Data expressed as mean ± SE. Values within rows with different superscripts differ significantly (P < 0.05)*.

**Table 4:** Evaluation of an experimental subunit vaccine (ESV) against enterotoxigenic *E. coli* (ETEC) on diarrhea scores of weanling piglets displaying colibacillosis.

**Days post-challenge**	**Score**	**Control**	**ESV**
1	1	15/15 (100%)	15/15 (100%)
2	1	15/15 (100%)	15/15 (100%)
3	3	11/15 (73.33%)	0/15 (0%)^*^
4	3	11/15 (73.33%)	3/15 (20%)^*^
5	2	2/15 (13.33%)	1/15 (6.66%)
6	1	15/15 (100%)	15/15 (100%)
7	1	15/15 (100%)	15/15 (100%)
8	1	15/15 (100%)	15/15 (100%)
9	1	15/15 (100%)	15/15 (100%)
10	1	15/15 (100%)	15/15 (100%)

*Experiment 3. Data are expressed as the number of piglets with diarrhea score/number of piglets in the group (%). Diarrhea score: 1, well-formed feces; 2, mild diarrhea; 3, severe diarrhea*.

* *Asterisk within rows indicates a significant difference at P < 0.001*.

**Figure 3:**
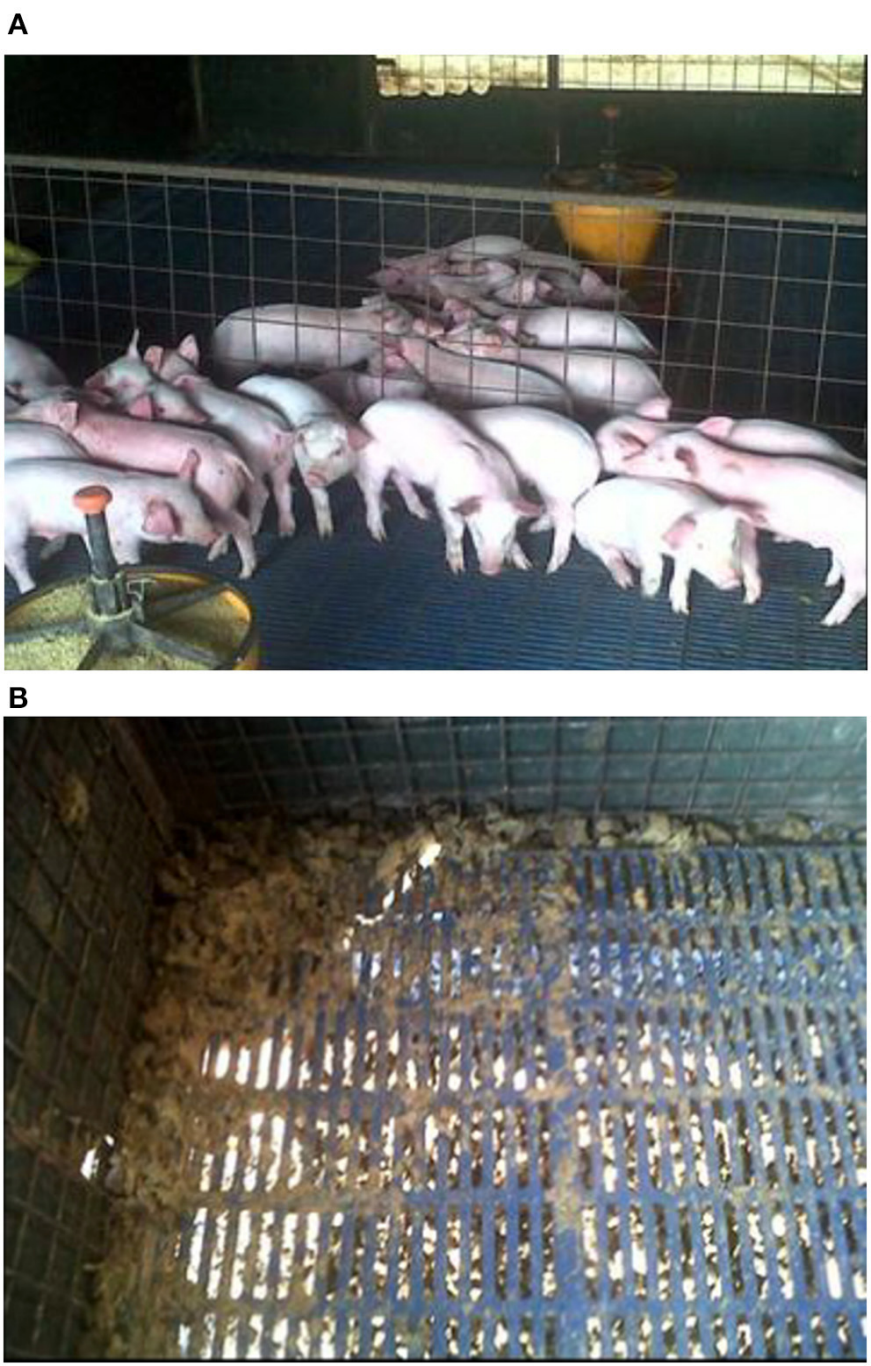
Visual representation of colibacillosis clinical signs and associated diarrhea in Experiment 3. **(A)** Piglets in the ESV vaccinated group displayed normal behavior and were absent of clinical signs (bottom) while piglets in the Control group huddled together and were lethargic (top). **(B)** Widespread colibacillosis was present in the Control group.

The results of the evaluation of ESV against ETEC on diarrhea scores of weanling piglets displaying colibacillosis in Experiment 3 are shown in Figure 3 and Table 5. A significant reduction (*P* < 0.001) in the percentage of ETEC associated diarrheas in the group treated with ESV compared to the saline control group at 72 h and 96 h post*-*ETEC challenge (Table 5). In the control group, piglets displayed sunken flanks and were lethargic, with many showing a roughened hair coat (hirsute) (Figure 3). Fecal scores for both treatment groups returned to normal by the 7th-day post-challenge. However, piglets in the ESV treated group had a significant (*P* < 0.05) increase in final body weight and body weight gain when compared to the saline treated controls (Table 5). No mortality was observed in either treatment group.

**Table 5:** Evaluation of an experimental subunit vaccine (ESV) against enterotoxigenic *E. coli* (ETEC) on initial body weight, final body weight (day 57), and body weight gain of weanling piglets.

	**Control**	**ESV**
Initial body weight	8.93 ± 0.13^a^	8.88 ± 0.09^a^
Final body weight	21.88 ± 0.35^b^	22.96 ± 0.22^a^
Body weight gain	12.95 ± 0.37^b^	14.07 ± 0.25^a^

*Experiment 3. Data expressed as mean ± SE. Values within rows with different superscripts differ significantly (P < 0.05)*.

## Discussion

Gastrointestinal illnesses with ETEC persist as a severe health issue for humans, piglets, and calves. In neonate and weaned piglets, ETEC-related diarrhea causes significant economic losses to the pig industry due to morbidity, mortality, and reduced performance and medication costs associated with the disease (15, 30). The continued emergence of antibiotic resistance among ETEC isolates associated with colibacillosis and post-weaning diarrhea has forced the scientific community to evaluate the use of alternative disease control measures, such as new vaccination strategies with oral fimbria adhesins, dietary administration zinc, spry-dried plasma, probiotics, prebiotics, organic acid, and even yolk antibodies from hens immunized with fimbria adhesins. In many instances, these new alternatives need further research and implementation (4).

In general, whole-cell vaccines, either attenuated or killed, may contain detrimental antigens associated with adverse effects or reduce efficacy. Furthermore, attenuated vaccines risk a reversion to a virulent from due to horizontal gene transfer from related Enterobacteriaceae. Killed vaccines (bacterins) require several immunizations and often do not confer serotype cross-protection. In contrast, subunit vaccines are developed to carry specific and well-defined epitopes that induce specific immune responses (25). Several successful studies have shown the promise of subunit vaccines against ETEC (31) or extraintestinal pathogenic *Escherichia coli* (32, 33) in humans. In mice, subunit vaccines against enteropathogenic *Escherichia coli* (EPEC) have been shown to elicit strong secretory IgA and cellular immune responses following nasal application (34). In swine, subunit vaccines have also shown positive results against porcine transmissible gastroenteritis virus (35), porcine reproductive and respiratory syndrome virus (30), and porcine epidemic diarrhea virus (36). In all cases, the successful subunit vaccines induce strong mucosal immunity. As mention previously, mucosal surfaces serve as the primary portal of entry for most pathogens. Hence, mucosal vaccination provides a safe and efficient mechanism to induce systemic and mucosal immunity against pathogens (37).

In the present study, intranasal vaccination with the ESV significantly increased the secretory IgA levels in the colostrum of postpartum sows in two independent trials compared to a commercial intramuscular vaccine, suggesting that mucosal immunity is activated with the ESV. It is remarkable to observed that intranasal vaccination, also mounted a similar IgG response to the IM vaccinated group. Vaccination of sows and the subsequent passive immunization of piglets against colibacillosis provided by antibodies are essential in preventing disease in suckling piglets (38). Further studies to evaluate the impact of the ESV antigen-specific colostrum antibody on the passive prevention of colibacillosis in matched suckling piglets require further investigation. Moreover, the results of two separate ETEC challenge experiments in the commercial neonate and weanling piglets that received two doses of ESV showed reductions of clinical symptoms associated with *E. coli* as well as significant reductions in the severity of *E. coli* associated diarrheas. In Experiment 2, the saline treated control neonatal suckling piglets experiencing diarrhea while the vaccinated group did not display diarrhea. Early vaccination and immune response are necessary to prevent post-weaning diarrhea (39). In Experiment 3, ESV protected ETEC induced colibacillosis after piglets have been weaned as well and the symptoms and the diarrhea were also significantly reduced. These results suggest that the inactivated orally administered subunit vaccine platform offers a promising alternative for controlling infections and pathogens associated with colibacillosis. Since the vaccine antigen is not based on fimbriae or toxins, ESV potentially offers universal colibacillosis protection that is simple to administer and safe to use.

## Data Availability Statement

The original contributions presented in the study are included in the article/supplementary material, further inquiries can be directed to the corresponding author/s.

## Ethics Statement

This animal study was reviewed and approved by in each experiment, all animal handling procedures followed the Institutional Committee's guidelines of use and care of experimental animals of the National Institute of Agronomic Technologies (INTA).

## Author Contributions

MJ and SL: conceptualization, methodology, and software. MJ and EG: visualization, investigation, and data curation. SL and JH: supervision and writing—original draft preparation. SL, JH, and XH-V: reviewing and editing. All the authors reviewed, edited, and approved the manuscript.

## Conflict of Interest

MJ, EG, JH, and SL are employed by Vetanco USA and Vetanco S.A. The authors declare that this study received funding from Vetanco USA Inc and Vetanco S.A. In this respect, the funder had the following involvement with the study: Study design, analysis, interpretation of the data, and writing. The remaining author declares that the research was conducted in the absence of any commercial or financial relationships that could be construed as a potential conflict of interest.
